# Novel prodrugs of decitabine with greater metabolic stability and less toxicity

**DOI:** 10.1186/s13148-019-0709-y

**Published:** 2019-08-01

**Authors:** Naoko Hattori, Magoichi Sako, Kana Kimura, Naoko Iida, Hideyuki Takeshima, Yoshitaka Nakata, Yutaka Kono, Toshikazu Ushijima

**Affiliations:** 1 Division of Epigenomics, National Cancer Center Research Institute, 5-1-1 Tsukiji, Chuo-ku, Tokyo, Japan; 2 Research and Development Division, Pharmaceutical Research Center, OHARA Pharmaceutical Co., Ltd., Koga, Japan

**Keywords:** Cancer therapy, DNMT inhibitor, Decitabine, DNA methylation, Epigenetics

## Abstract

**Background:**

DNA demethylation therapy is now used in practice for hematological tumors and is being developed for solid tumors. Nevertheless, it is difficult to achieve stable pharmacokinetics with the current DNA-demethylating agents, azacitidine (AZA) and decitabine (DAC), because of their rapid deamination by cytidine deaminase in vivo and spontaneous hydrolytic cleavage. Here, we aimed to develop metabolically stable prodrugs of AZA and DAC as novel DNA-demethylating agents.

**Results:**

Thirty-five 5′-O-trialkylsilylated AZAs/DACs were synthesized with potential resistance to deamination. Out of these, 11 compounds exhibited demethylating activity similar to that of DAC and guadecitabine, and a suitable aqueous solubility. Pharmacokinetic analysis in mice showed that OR-2003 displayed the highest serum concentration and the area under the curve in an intraperitoneal experiment, whereas OR-2100 exhibited high stability to cytidine deaminase. Treatment of cells with OR-2003 and OR-2100 depleted DNA methyltransferase 1 completely and induced both gene-specific and genome-wide demethylation. The treatment suppressed the growth of multiple types of cancer cells and induced re-expression of tumor suppressor genes. The anti-tumor effect and DNA demethylation effect of OR-2003 and OR-2100 were comparable to that of DAC with fewer adverse effects in vivo.

**Conclusions:**

We developed two novel prodrugs of DAC that exhibited greater stability, comparable DNA demethylation activity, and less toxicity. These compounds are expected to overcome the difficulty in achieving stable pharmacokinetics in patients, leading to maximum DNA demethylation activity with minimum adverse effects.

**Electronic supplementary material:**

The online version of this article (10.1186/s13148-019-0709-y) contains supplementary material, which is available to authorized users.

## Background

Epigenetic changes, including DNA methylation alteration, are known to be associated with the development and progression of tumors. This realization has brought the use of DNA-demethylating agents as a therapeutic approach into practice for hematological tumors, and a large number of clinical trials are being conducted for solid tumors [1–3]. Two DNA-demethylating agents, namely azacitidine (AZA, 5-azacytidine) and decitabine (DAC, 2′-deoxy-5-azacytidine), have been approved by the Food and Drug Administration for treating myelodysplastic syndrome (MDS) and acute myeloid leukemia (AML) [4, 5]. Upon incorporation into the genomic DNA, these are covalently bound to DNA methyltransferase 1 (DNMT1) on the occasion of maintenance methylation [6]. The DNMT1 irreversibly bound to a DNA strand is recognized as a DNA adduct that gets excised by the base excision repair and finally degraded by proteasomes [7, 8]. The degradation of DNMT1 depletes cellular functional DNMT1, causing DNA demethylation over cell cycles (passive demethylation) and inducing re-expression of silenced tumor-suppressor genes [9].

Based upon this mechanism of action, a therapeutically effective DNA-demethylating agent needs to be incorporated into the DNA of tumor cells at a concentration that can decrease DNMT1 and allow cell cycle progression. Excessive doses of DNA-demethylating agents can cause extensive DNA damage and induce cell cycle arrest [10]. In contrast, lower doses will retain the functional DNMT1, rendering the demethylation action ineffective. A serious limitation associated with the administration of suitable doses of DNA-demethylating agents is their poor in vivo stability owing to deamination by ubiquitously present cytidine deaminase and spontaneous hydrolytic cleavage of the triazine ring [11]. To overcome this problem, several DNA-demethylating agents are now being developed using short oligonucleotides or as oral formulations; some of these are in clinical trials [12–15].

In the present study, we designed 35 5′-O-trialkylsilylated AZAs and DACs considering the fact that enzymatic cleavage of the silylated functional group has not yet been reported. Using a high-throughput assay system that we previously developed, we readily detected the demethylation of a sensitive marker promoter CpG island by enhanced green fluorescent protein (EGFP) fluorescence and luciferase activity [16]. Subsequent analyses of in vitro efficacy, pharmacokinetics, and in vivo efficacy and toxicity showed promising utility of two compounds.

## Results

### Isolation of potent, hydrophilic, and disparate stability compounds

**Fig. 1:**
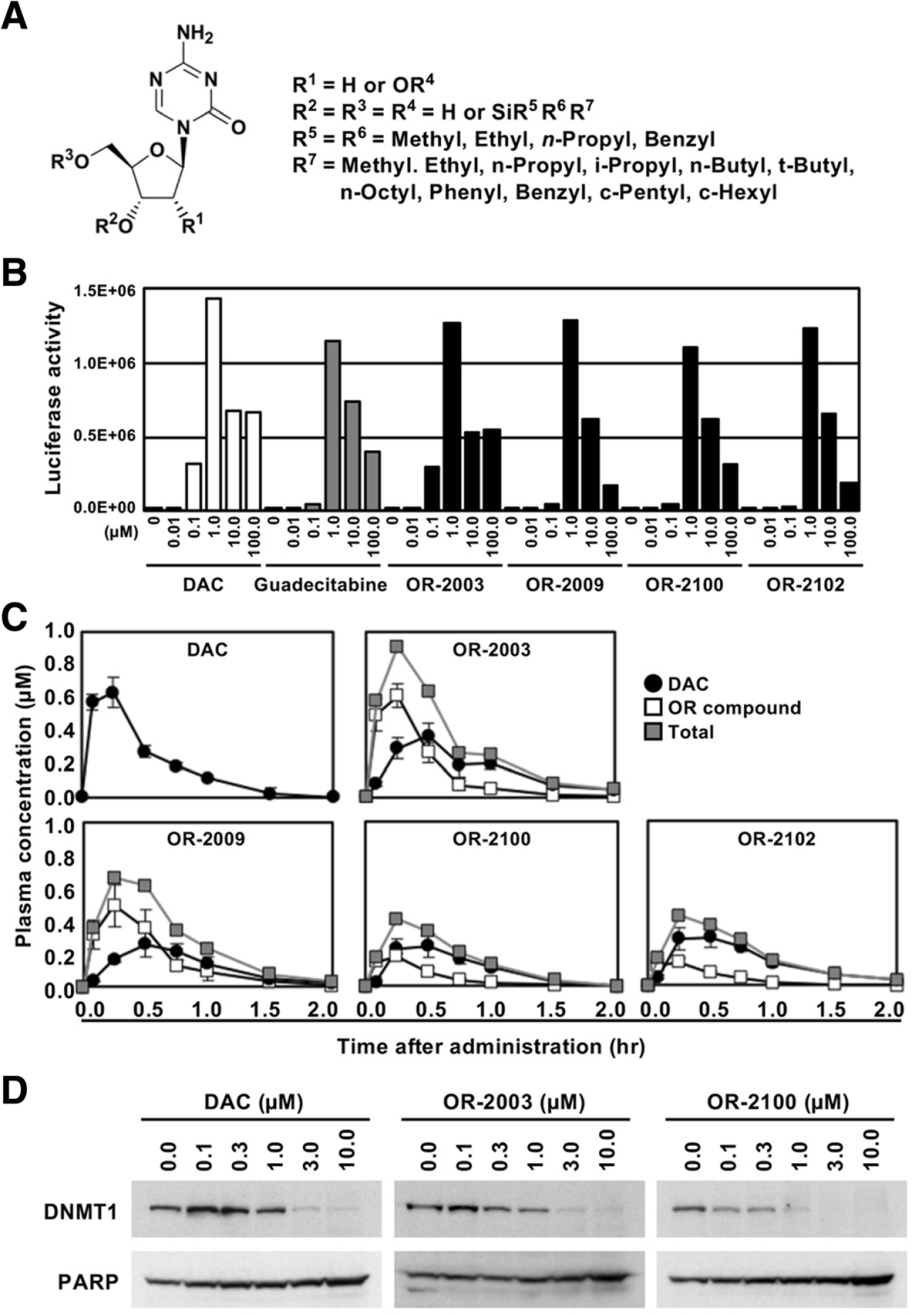
Synthesis and isolation of hydrophilic compounds with disparate stability. **a** Structures of 5′-O-trialkylsilylated AZAs or DACs. A variety of 5′-O-trialkylsilylated DACs were synthesized that were potentially resistant to deamination by cytidine deaminase and spontaneous hydrolytic cleavage of the triazine ring. **b** Luciferase activity of DAC, guadecitabine, and four compounds. From the 5′-O-trialkylsilylated DACs that showed a strong luciferase activity, OR-2003, OR-2009, OR-2100, and OR-2102 were selected. **c** Comparison of plasma concentrations of the four compounds with DAC. Pharmacokinetics of the four compounds were analyzed using mice administered a compound at the same molar concentrations as DAC. **d** Depletion of DNMT1 by DAC, OR-2003, and OR-2100. Levels of DNMT1 protein in cells treated with a compound were analyzed using western blotting. The DNMT1 protein showed a dose-dependent decrease, and at doses higher than 3 μM, DNMT1 was depleted

Thirty-five different 5′-O-trialkylsilylated AZAs and DACs were synthesized, which were resistant to deamination by cytidine deaminase and spontaneous hydrolytic cleavage of the triazine ring (Fig. 1a, Additional file 1: Supplementary Materials). The DNA-demethylating activity of these compounds was screened by measuring the luciferase activity in HML58-3 cells, which had been engineered to have a luciferase reporter under a sensitive methylated marker promoter to detect DNA demethylation activity [16]. In this screening assay, no luciferase activity was observed upon treating cells with any of the 10 5′-O-trialkylsilylated AZAs. However, treatment with 1.0 μM of 11 of the 5′-O-trialkylsilylated DACs resulted in a luminescence of 1.0 × 10^6^ counts/photons per second (cps) or more (Fig. 1b for representative compounds, and Additional file 1: Figure S1A and B for all compounds). None of the compounds showed a dose-dependent increase in luminescence, and all of them exhibited an optimal dose for the maximum luciferase activity.

For the 11 compounds with sufficient luciferase activity, we analyzed the partition coefficients (log *P* values) using the shake-flask method [17], and their half-lives, which were expected to be similar to the time to release of their metabolite, namely DAC (Additional file 1: Figure S1B). All compounds except OR-2001 had higher log *P* values (from 1.48 to 4.15) than DAC (log *P* = − 0.32), suggesting their feasibility as oral formulations (Additional file 1: Figure S1B). For further analysis, we selected OR-2003 as the compound with the shortest half-life (1 h) and OR-2009, OR-2100, and OR-2102 as compounds with the longest half-lives (≥ 14 h) to release DAC almost quantitatively (Table 1).

**Table 1:** Structure, log *P* value, stability, and pharmacokinetics of DAC and 5′-O-trialkylsilylated DACs

		DAC	OR-2003	OR-2009	OR-2100	OR-2102
	*R* ^1^	H	H	H	H	H
	*R* ^2^	H	H	H	H	H
	*R*^3^ = *R*^4^	H	H	H	H	H
	*R* ^5^	–	Methyl	Methyl	Ethyl	Ethyl
	*R* ^6^	–	Methyl	Methyl	Ethyl	Ethyl
	*R* ^7^	–	*n*-Propyl	*c*-Hexyl	Ethyl	*n*-Propyl
Log *P* value		− 0.32	1.64	2.52	2.14	2.54
Stability	t_1/2_ in PBS	20 h*	20** + 1 h	20** + 14 h	20** + 16 h	20** + 19 h
	Enzymatic	Complete degradation after 0.5 h	Retain 52% after 1 h	Retain 75% after 2 h	Retain 84% after 2 h	Retain 46% after 2 h
AUC _0 → lim_ (μM·hr)	OR compound	—	0.305	0.314	0.168	0.081
	DAC	0.378	0.338	0.238	0.266	0.285
	Total	0.378	0.643	0.552	0.434	0.366

*DAC* decitabine, *PBS* phosphate-buffered saline

*Reference: Yoo C.B. et al. Cancer Research 2007; 67:6400–6408

**20 h: t_1/2_ in PBS of DAC

To analyze the pharmacokinetics and membrane permeability of the four OR compounds and their metabolite DAC, plasma concentrations were measured after their intraperitoneal administration to mice at the same molar concentrations as DAC. All compounds including DAC reached the maximum plasma concentration of administered compounds themselves (*C*_max_) within 15 min. Moreover, OR-2003 exhibited the highest *C*_max_ among the four OR compounds (Fig. 1c). The peak time of metabolite DAC of the four OR compounds was calculated to be approximately 30 min, suggesting a rapid in vivo metabolism of the compounds. With respect to the total area under the curve (AUC) of the OR compounds and their metabolite DAC, OR-2003, OR-2009, and OR-2100 had larger AUCs than that of DAC, while OR-2102 did not.

Resistance to deamination by cytidine deaminase is important for the development of a prodrug of DAC, and the stability of the four OR compounds was analyzed under the presence of cytidine deaminase (CDA) (Table 1). Although DAC resulted in complete degradation after 0.5 h, OR-2003 and OR-2100 showed good retention of 52% after 1 h and 84% after 2 h, respectively. Because of the disparate features of metabolic processes in vitro and in vivo, OR-2003 and OR-2100 were selected as candidates for novel prodrugs of DAC. Their mode of action was confirmed by analyzing the degradation of DNMT1. The DNMT1 protein levels decreased in a dose-dependent manner for the two OR compounds; for DAC, DNMT1 was depleted at 3 and 10 μM in HML58-3 cells (Fig. 1d).

### Gene-specific and genome-wide DNA demethylation by OR-2003 and OR-2100

**Fig. 2:**
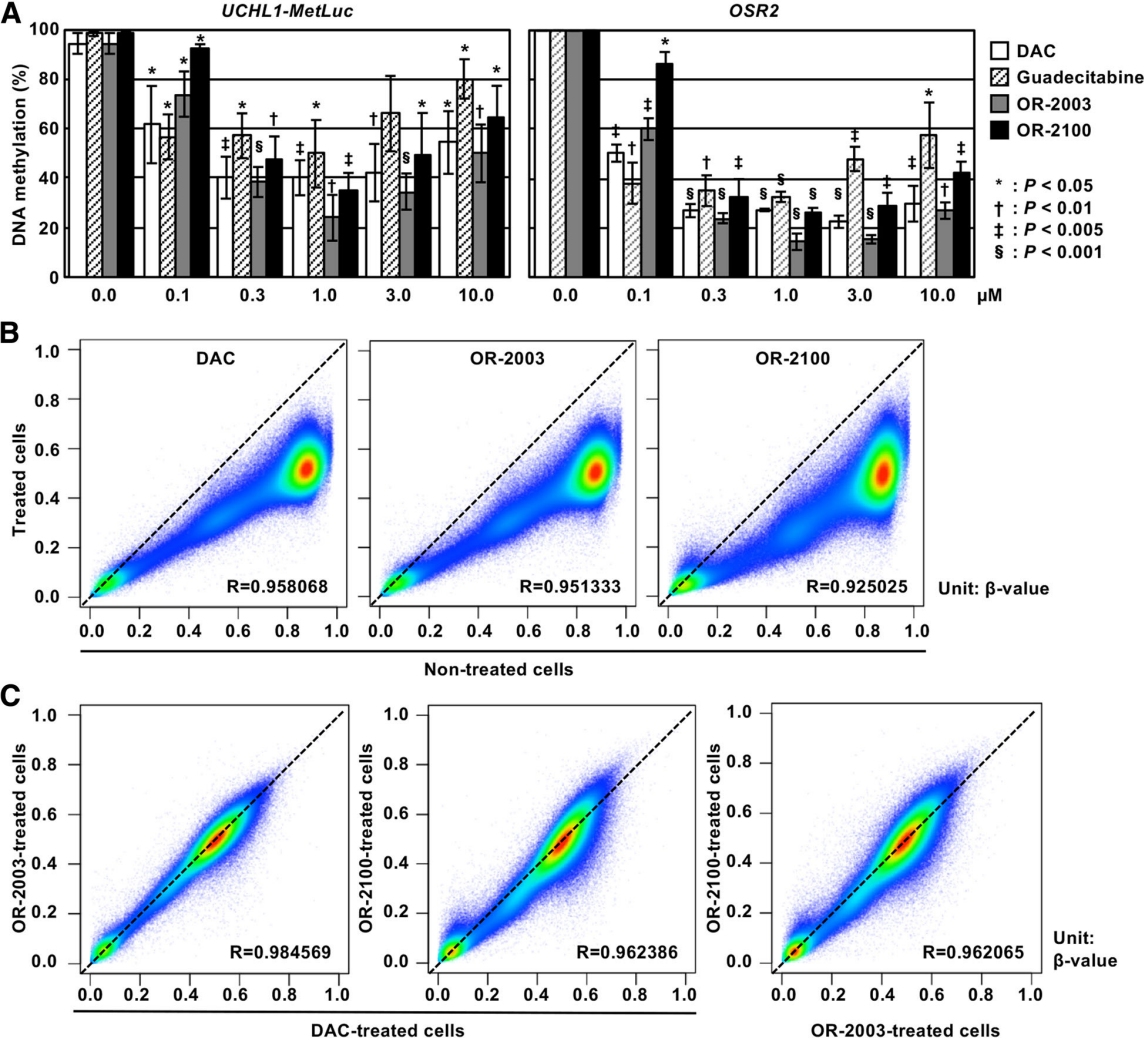
Effects of OR-2003 and OR-2100 on DNA methylation. **a** DNA demethylation of marker genes by OR-2003 and OR-2100. Methylation levels of an exogenous *UCHL1* promoter CpG island and an endogenous *OSR2* promoter CpG island were analyzed by qMSP in drug-treated cells (0.0 to 10.0 μM). OR-2003 induced demethylation of these two genes at levels similar to or stronger than that of DAC and that of guadecitabine. OR-2100 induced a demethylation effect similar to or a slightly lower than that of DAC, and stronger than that of guadecitabine. Each qMSP analysis was performed thrice, and the results are represented as mean ± SD. **b** Genome-wide DNA demethylation by OR-2003 and OR-2100. Genome-wide DNA methylation levels were analyzed using an Infinium Human MethylationEPIC BeadChip in non-treated cells and drug-treated cells (1 μM) and compared. Methylation levels are shown as *β*-values (0.0 to 1.0). Comparison of 865,918 CpG sites between non-treated cells and treated cells showed that both OR-2003 and OR-2100 induced strong DNA demethylation. **c** Comparison of demethylated regions among three compounds. The demethylated regions were very similar between DAC and OR-2003, whereas these differed slightly between DAC and OR-2100 and between OR-2003 and OR-2100

To analyze the effect of OR-2003 and OR-2100 on DNA demethylation, we first analyzed the DNA methylation levels of (1) the marker region of our detection system, namely exogenous *UCHL1* promoter (Additional file 1: Figure S2) and (2) a promoter CpG island of a gene frequently methylated in cancer cells, namely the *OSR2* promoter CpG island, using quantitative MSP [18]. OR-2003 induced demethylation of the two genes as strongly as DAC whereas OR-2100 induced a slightly lower demethylation (Fig. 2a). Compared to guadecitabine, OR-2003 and OR-2100 showed overall stronger demethylation effects at the two genes. The two OR compounds, DAC, and guadecitabine, exerted the strongest effect of DNA demethylation at 1.0 μM. It is known that high doses of DNA-demethylating agents induce cell-cycle arrest and fail to induce DNA demethylation. Since guadecitabine displayed a weaker demethylation effect than OR-2003 and OR-2100 in vitro, DAC was used as a control agent for further experiments.

Genome-wide DNA methylation analysis using Infinium Human MethylationEPIC BeadChip showed both OR-2003 and OR-2100 induced as strong DNA demethylation as DAC in HML58-3 cells (Fig. 2b). OR-2100 induced a larger variation in *β*-values at regions highly methylated in non-treated cells, suggesting that the demethylating ability of OR-2100 could vary among genomic regions or individual cells. The correlation analysis between cells treated with different compounds showed that the demethylated genomic regions were similar between DAC and OR-2003, whereas these differed slightly between DAC and OR-2100 (Fig. 2c).

### In vitro tumor-suppressive activity and epigenetic reprogramming ability of OR-2003 and OR-2100

**Fig. 3:**
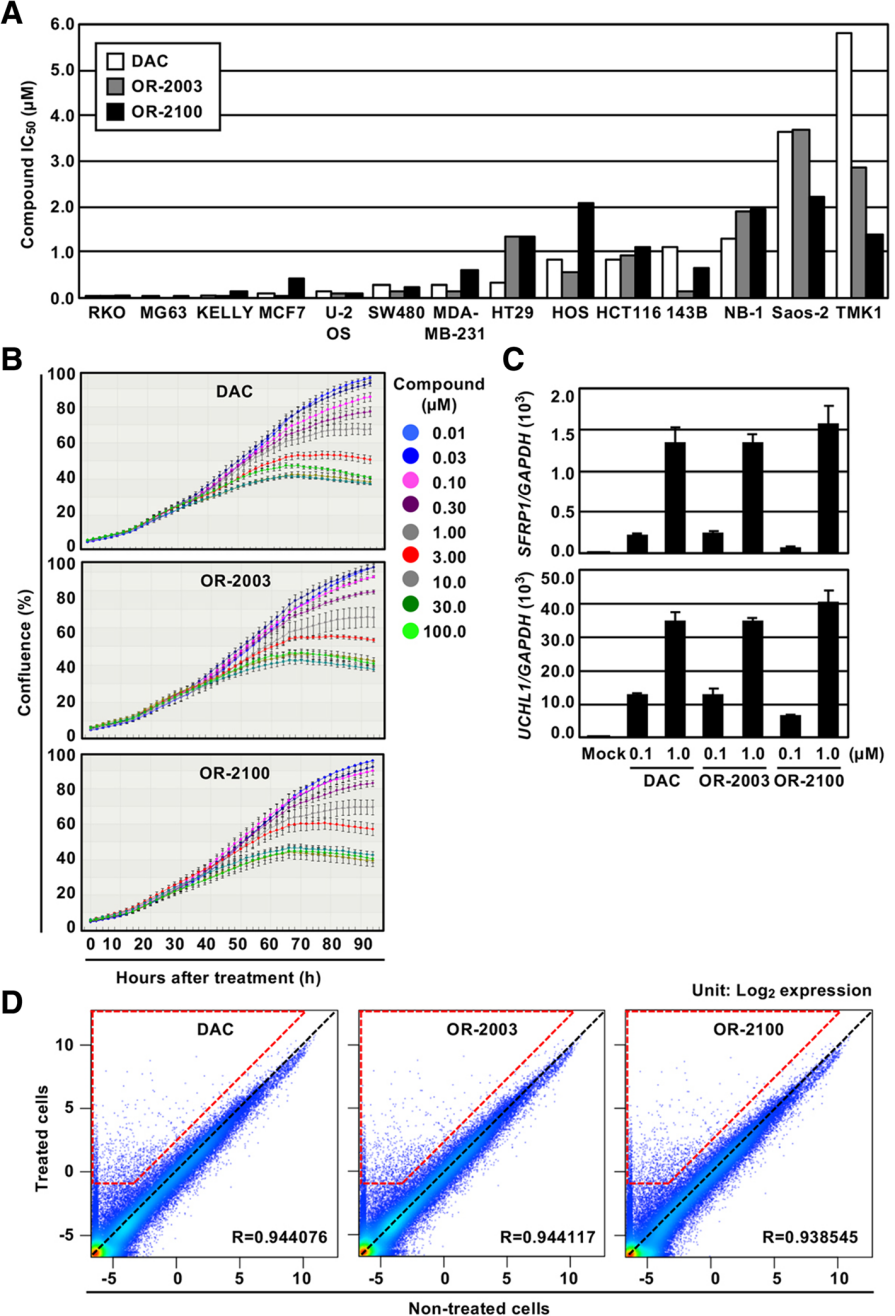
Effects of OR-2003 and OR-2100 on in vitro tumor-suppressive activity. **a** Cell growth inhibition ratio. The IC_50_ values (μM) of the compounds were analyzed after the treatment using multiple types of cancer cell lines. Although the IC_50_ value varied among the cell lines, almost identical values were observed for DAC, OR-2003, and OR-2100. **b** Change in cell growth after treatment of compounds. The effect on growth of HCT116 cells was analyzed by monitoring their growth for 94 h using IncuCyte. OR-2003 and OR-2100 showed a decrease in the cell number similar to that of DAC. Results are shown as mean ± SD. **c** Reactivation of two tumor-suppressor genes. The re-expression of tumor-suppressor genes known to be methylated in HCT116 cells was analyzed. Both *SFRP1* and *UCHL1* showed re-expression in a dose-dependent manner. Each RT-qPCR analysis was performed thrice and the results are presented as mean ± SD. **d** Induction of gene expression by OR-2003 and OR-2100, along with DAC. Expression levels (log_2_ values) of 58,341 probes obtained by an expression microarray were compared for non-treated cells (*x*-axis) and drug (1 μM)-treated cells (*y*-axis). Probes with no change were plotted on the black dashed line, and the genes upregulated more than twofold and to − 1.0 log_2_ value by the drug treatment are surrounded by the trapezoid with red dashed lines. The numbers for DAC, OR-2003, and OR-2100 were 2011, 2060, and 2041, respectively

Tumor-suppressive activity of OR-2003 and OR-2100 was first analyzed in vitro by observing their effects on the growth of multiple types of cancer cell lines, including colorectal cancer, breast cancer, gastric cancer, sarcoma, and neuroblastoma (Fig. 3a). The IC_50_ values of DAC, OR-2003, and OR-2100 varied among the cell lines from 0.2 nM to 5.8 μM. In RKO cells, the most sensitive cell line to DAC, the IC_50_ values were 0.20 nM, 0.04 μM, and 0.06 μM, respectively. In TMK1 cells, the most resistant cell line to DAC, they were 5.83 μM, 2.84 μM, and 1.41 μM, respectively. In HCT116 cells, the IC_50_ values of DAC, OR-2003, and OR-2100 were 0.85, 0.91, and 1.11 μM, respectively. DAC suppressed the cell growth in a dose-dependent manner, and the two OR compounds exhibited similar growth-suppressive effects in HCT116 cells (Fig. 3b).

To confirm the induction of epigenetic reprogramming, demethylation of tumor-suppressor genes, known to be methylation-silenced in HCT116 cells and readily demethylated by DNA-demethylating agents, was analyzed [19, 20]. Both *SFRP1* and *UCHL1* showed re-expression in a dose-dependent manner, implying induction of epigenetic reprogramming (Fig. 3c). We performed a genome-wide gene expression analysis using expression microarray and found that both OR-2003 and OR-2100 induced re-expression of a large number of genes as DAC in HML58-3 (Fig. 3d). A pathway analysis showed that the top three pathways enriched for DAC-, OR-2003-, and OR-2100-treated cells were identical and one additional pathway was shared (Table 2), indicating that almost identical gene sets were re-expressed by these drugs and that epigenetic reprogramming ability of OR-2003 and OR-2100 was comparable to that of DAC.

**Table 2:** Top five enriched canonical pathways in drug-treated cells compared with mock-treated cells

Drug		Ingenuity canonical pathways	*P* value	Overlapping rate (%) (enriched gene no./total no.)
DAC	1	Interferon signaling	4.47E−07	38.2% (13/34)
	2	Hepatic fibrosis/hepatic stellate cell activation	2.68E−05	16.7% (30/180)
	3	TREM1 signaling	5.79E−04	20.0% (14/70)
	4	Activation of IRF by cytosolic pattern recognition receptors	1.02E−03	20.7% (12/58)
	5	Granulocyte adhesion and diapedesis	1.06E−03	14.7% (24/163)
OR-2003	1	Interferon signaling	3.72E−06	35.3% (12/34)
	2	Hepatic fibrosis/hepatic stellate cell activation	3.23E−05	16.7% (30/180)
	3	TREM1 signaling	6.39E−04	20.0% (14/70)
	4	Role of macrophages, fibroblasts, and endothelial cells in rheumatoid arthritis	1.02E−03	12.8% (38/298)
	5	Activation of IRF by cytosolic pattern recognition receptors	1.12E−03	20.7% (12/58)
OR-2100	1	Interferon signaling	4.40E−07	38.2% (13/34)
	2	Hepatic fibrosis/hepatic stellate cell activation	6.66E−05	16.1% (29/180)
	3	TREM1 signaling	5.70E−04	20.0% (14/70)
	4	Activation of IRF by cytosolic pattern recognition receptors	1.01E−03	20.7% (12/58)
	5	Role of macrophages, fibroblasts, and endothelial cells in rheumatoid arthritis	1.56E−03	12.4% (37/298)

**Fig. 4:**
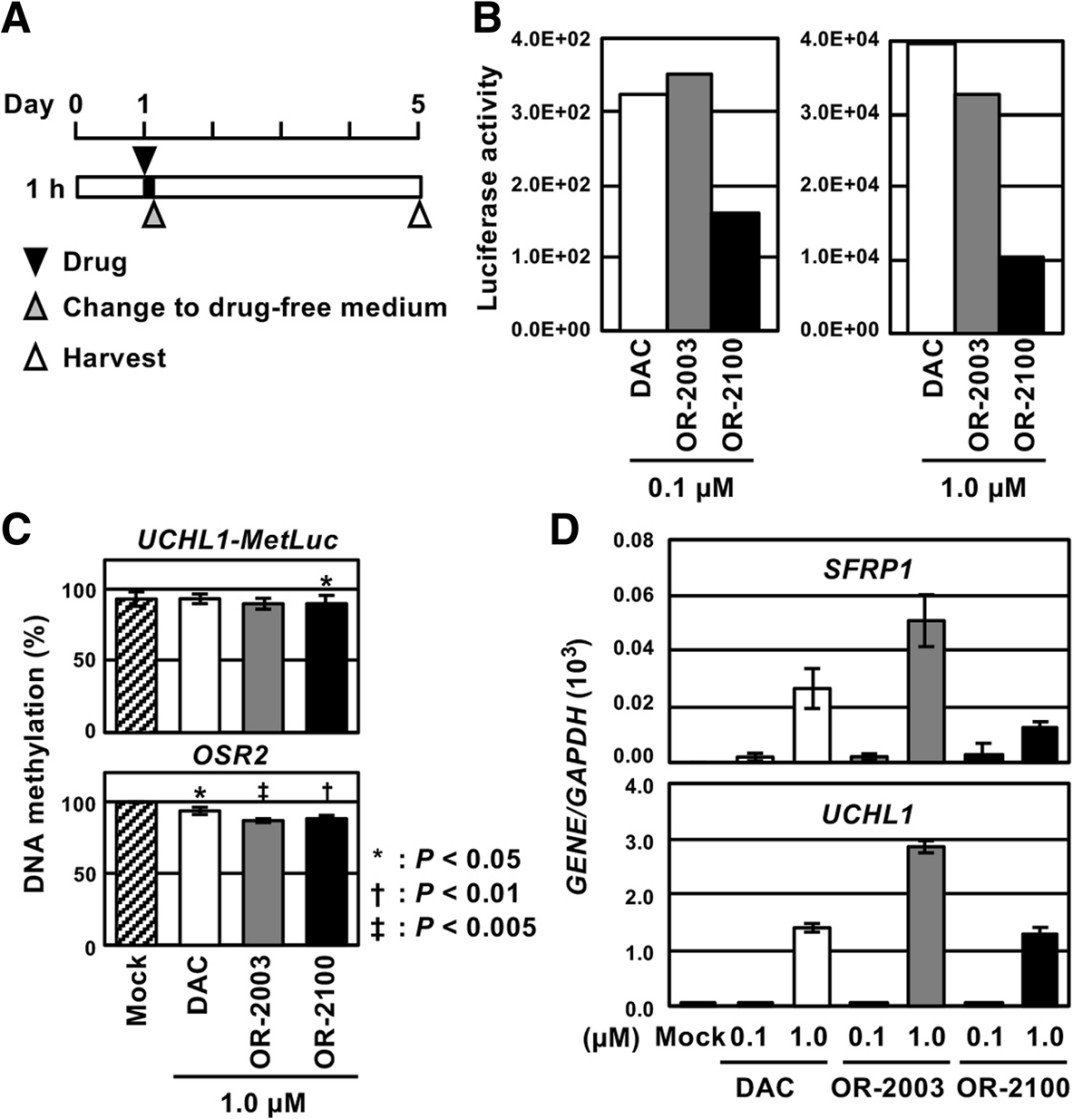
Effect of a pulse exposure of OR-2003 and OR-2100. **a** Treatment schedule of the pulse exposure analysis. HML58-3 cells were treated with 0.1 and 1.0 μM of DAC, OR-2003, and OR-2100, and the compounds were removed at 1 h by replacing the medium with drug-free medium. The effects were analyzed 5 days after the drug administration. **b** Luciferase activity due to marker gene demethylation. OR-2003 showed luciferase activity similar to that of DAC, whereas OR-2100 exhibited a luciferase activity weaker than that of DAC. **c** DNA demethylation of an exogenous marker gene and endogenous gene. Methylation levels were analyzed by qMSP in the drug-treated cells (1.0 μM). OR-2003 and OR-2100 induced stronger demethylation of these two genes compared with that induced by DAC. Values show mean ± SD of three experiments. **d** Reactivation of tumor-suppressor genes. The higher dose (1.0 μM) of all compounds induced expression of *SFRP1* and *UCHL1.* The expression level induced by OR-2003 was twice as high as that induced by DAC. Each RT-qPCR analysis was performed thrice, and the results are represented as mean ± SD

To analyze the efficacy of cellular uptake of OR-2003 and OR-2100, a pulse exposure experiment was conducted. We exposed HML58-3 cells to 0.1 and 1.0 μM of these two compounds for 1 h (Fig. 4a), and DNA-demethylating activity and re-expression of tumor-suppressor genes were analyzed on day 5. OR-2003, but not OR-2100, displayed luciferase activity comparable to that of DAC (Fig. 4b) and a stronger induction of endogenous tumor-suppressor genes, *SFRP1* and *UCHL1* (Fig. 4d). These data showed that OR-2003 could be rapidly taken into cells and readily converted into DAC, leading to an activity equal to or stronger than that of DAC.

### In vivo anti-tumor effect of OR-2003 and OR-2100

**Fig. 5:**
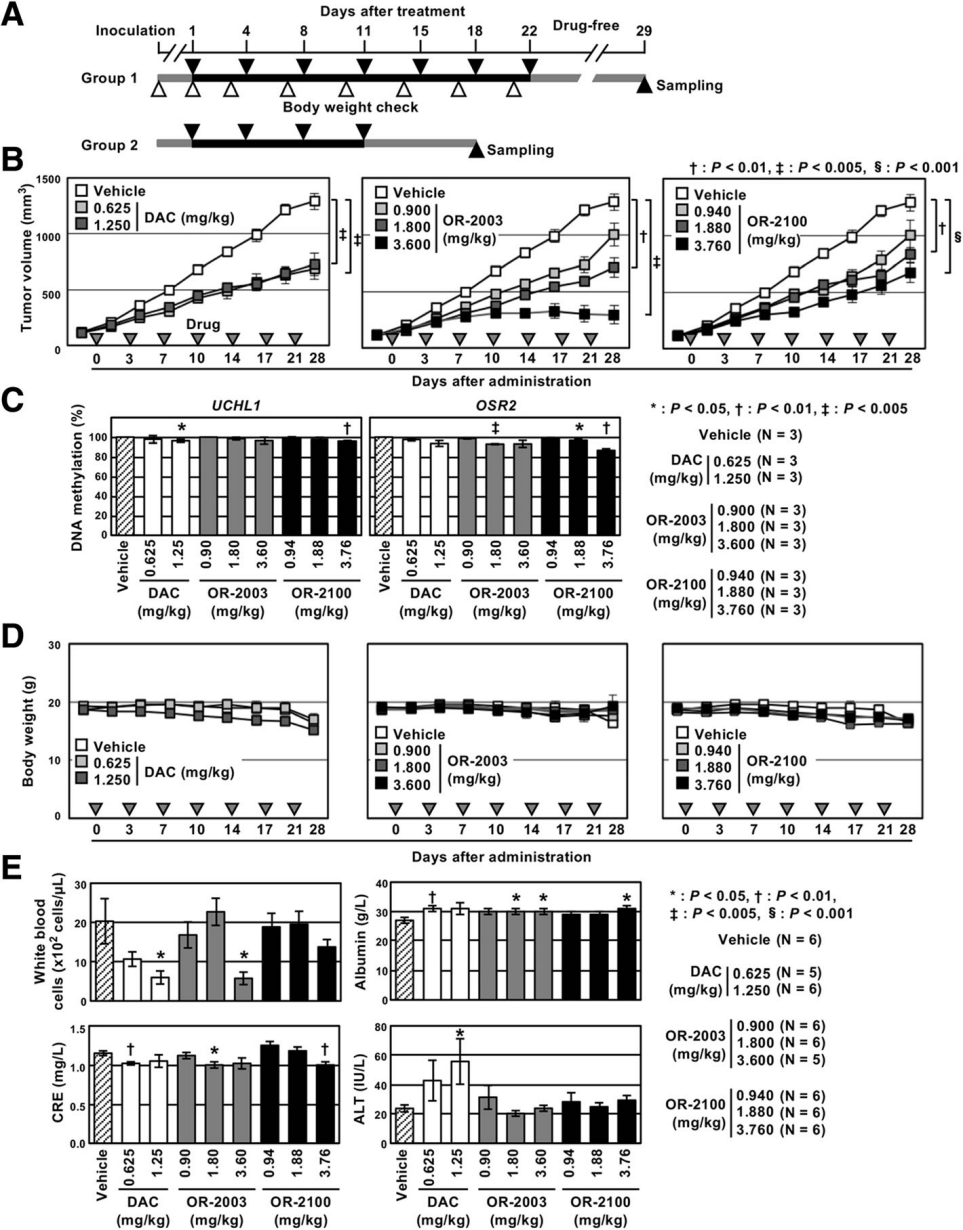
In vivo anti-tumor effects of OR-2003 and OR-2100. **a** Timing and duration of the treatment. HCT116 cells were subcutaneously injected into nude mice, and once tumors reached an average volume of 80 mm^3^, mice were treated by intraperitoneal injection of vehicle, DAC, OR-2003, or OR-2100 twice per week. The mice in group 1 and group 2 were treated with drugs for 22 days and 11 days, respectively. **b** Changes in tumor volumes. The tumor volume was measured twice per week for mice in group 1. DAC (0.625 and 1.250 mg/kg) suppressed the xenograft growth, but dose dependence was unclear. Both OR-2003 and OR-2100 demonstrated significant and dose-dependent inhibition of tumor growth. **c** DNA demethylation in xenograft tumors. The effect of OR-2003 and OR-2100 on DNA demethylation was analyzed using xenograft tumors of mice in group 2. OR-2003 and OR-2100 induced significant demethylation in vivo comparable to DAC although the level was small. Values show mean ± SD of three experiments. **d** Body weight changes of mice in the three groups. There was no significant difference in the severity of body weight loss at the time of sacrifice among the three groups. Results are shown as mean ± SD. **e** Adverse-effects observed in the blood tests. A decrease in WBC counts and impairment of the liver function was observed in the group treated with DAC. On the other hand, OR-2003 and OR-2100 at concentrations equimolar to DAC exhibited no adverse effects on WBC counts and liver function. Results are shown as mean ± SD (*n* = 5 or 6/group)

Finally, in vivo anti-tumor effects of OR-2003 and OR-2100, along with DAC, were evaluated in mice bearing HCT116 xenografts (Fig. 5a). In our initial experiment, 7.50 and 3.75 mg/kg of DAC were administered according to a previous study on in vivo tolerability [13]. However, severe body weight loss was observed (data not shown). Thus, we changed the doses of DAC to 0.625 and 1.250 mg/kg and adopted the doses of OR-2003 and OR-2100 at the same molar concentrations as that of DAC (0.900 and 1.800 mg/kg for OR-2003, 0.940 and 1.880 mg/kg for OR-2100) and a twice higher dose (3.600 mg/kg for OR-2003 and 3.760 mg/kg for OR-2100).

DAC suppressed the xenograft growth; however, it was unclear whether this effect was dose-dependent. In contrast, both OR-2003 and OR-2100 demonstrated significant and dose-dependent inhibition of tumor growth; the highest dose (3.600 mg/kg) of OR-2003 produced the strongest effect (Fig. 5b). Xenograft tumors were collected from the mice of group 2 in the middle of drug treatment to observe the effect in cells responding to the drug (Fig. 5a), and we analyzed DNA demethylation levels of two promoters. OR-2003 and OR-2100 induced significant demethylation of the two promoters as DAC although the effect was small (Fig. 5c).

As for the adverse effects, there was no significant difference in the severity of body weight loss among the three groups (Fig. 5d). The white blood cell (WBC) count and liver function, assessed by aspartate aminotransferase (AST) and alanine aminotransferase (ALT) activities, were affected in the groups treated with DAC, even at a lower dose (Fig. 5e and Additional file 1: Figure S3). At doses equimolar to that of DAC, OR-2003 and OR-2100 exerted no influence on the WBC count and liver function, indicating less adverse effects compared with those of DAC. The highest dose of OR-2003 affected the WBC count (Fig. 5e). These findings demonstrated that OR-2003 exerted an anti-tumor effect comparable to DAC but with less toxicity.

## Discussion

The present study identified two types of 5′-O-trialkylsilylated DACs, namely OR-2003 and OR-2100, as novel prodrugs of DAC. The in vivo pharmacokinetic analysis showed OR-2003 to have the highest *C*_max_ and AUC, whereas OR-2100 exhibited a high stability to cytidine deaminase. Both OR-2003 and 2100 showed in vivo dose-dependent inhibition of tumor growth and a DNA demethylation effect, supporting their good drug efficacy. In addition, a high dose of OR-2100 induced no adverse effects, and only a high dose of OR-2003 reduced the WBC counts. In contrast, a half molar concentration of DAC impaired the liver function, in addition to the reduction of the WBC count, indicating that both OR-2003 and OR-2100 have wider safety margins than DAC. This was considered because a prolonged exposure of tumor cells to DAC was achieved while keeping the maximum plasma concentration relatively low, thereby avoiding toxicity.

The 1-h pulse exposure experiment confirmed that OR-2100 exhibited a substantially weaker activity in this condition. In this experiment, the prodrugs need to enter cells during the 1-h exposure, and metabolite DAC needs to be constantly released until a cell enters into the S phase. Therefore, the experiment reflected both the cellular uptake and stability of the prodrugs. At the same time, in vivo pharmacokinetics analysis showed that both OR-2100 and OR-2003 had a similar release pattern of their metabolite DAC (Fig. 1c). Taken together, the much lower *C*_max_ of OR-2100 was considered to be due to its inefficient uptake by HCT116 cells (pulse exposure experiment) and peritoneal cells (pharmacokinetics analysis). At the same time, OR-2100 was capable of releasing its metabolite DAC with relatively low *C*_max_. Since myelotoxicity of DAC is considered to be dependent upon *C*_max_ [21], the effective release of DAC with low *C*_max_ may have helped avoiding toxicity with high efficacy.

The CpG sites demethylated by OR-2003 were highly consistent with those demethylated by DAC; however, those acted upon by OR-2100 were slightly different (Fig. 2c). The target regions of DNA demethylation are determined by the genomic structure and epigenetic modifications of individual genomic regions [22]. Since the drug-mediated cytotoxic stress is expected to change epigenetic modifications of several genomic regions, the degree or kind of cellular stress induced by OR-2100 might have been different from that induced by DAC. To support this speculation, less adverse effects were induced by OR-2100 than those by DAC (Fig. 5d).

## Conclusions

We identified two novel prodrugs of DAC with higher stability and less toxicity. Also, these compounds exhibited higher log *P* values than DAC, suggesting their application as oral formulations. The in vitro and in vivo findings in mice warranted further investigations to develop these compounds as novel DNA-demethylating agents.

## Methods

### Preparation of various types of 5′-O-trialkylsilylated azacitidines and decitabines

Different types of trialkylsilyl chlorides (1.3 mM) were added to a stirred solution of AZA or DAC (1.0 mM) and morpholine (3.0 mM) in dry *N*,*N*-dimethylacetamide (5.0 mL) at 0 °C. This solution was stirred for 1 h. The resulting reaction mixture was added to a stirred mixture of brine (10 mL) and ethyl acetate (50 mL) to extract the desired compound. The separated aqueous phase was extracted twice using ethyl acetate (25 mL). After collecting, the ethyl acetate phases were washed twice with brine (10 mL) and then dried over anhydrous magnesium sulfate, followed by condensation under reduced pressure. The resulting residual oil was subjected to silica gel column chromatography; it was eluted using a mixture of chloroform and methanol to obtain the desired 5′-O-trialkylsilylated AZAs or DACs in a pure state. The alkyl moieties of trialkylated silyl group were *R*^5^ = *R*^6^ = methyl, ethyl, *n*-propyl, or benzyl; *R*^7^ = ethyl, *n*-propyl, *i*-propyl, *n*-butyl, *t*-butyl, *n*-octyl, phenyl, benzyl, *c*-pentyl, and *c*-hexyl. Further information about 5′-O-trialkylsilylated AZAs and DACs is shown in Additional file 1: Supplemental Information.

### Partition coefficients (log *P* values) of 5′-O-trialkylsilylated decitabines

Log *P* values of the obtained 5′-O-trialkylsilylated DACs were calculated using the shake-flask method [17] with following conditions: 10 μL of each sample, 1.0 mg/100 μL of dimethyl sulfoxide (DMSO), 200 μL of *n*-octanol, and 200 μL of phosphate-buffered saline (PBS) solution. The method employed HPLC with the following conditions: ZORBAX Bonus C18 column (4.6 × 150 mm, 3 μm); eluent: *A* = 10 mM HCO_2_NH_4_, *B* = acetonitrile, *A*:*B* = 50:50 (an isocratic mode for 5 min), and *A*:*B* = 99:1–20:80 (a gradient mode for 15 min); flow rate = 1.0 mL/min; oven temperature = 40 °C; detection = 240 nm.

### Stability of 5′-O-trialkylsilylated decitabines in the presence of cytidine deaminase

Five microliters of DAC derivatives (1.0 mg/100 μL of DMSO) and 5 μL of a human recombinant cytidine deaminase [1–146aa, His-tagged, (AT Gen Co. Ltd.; Seoul, Korea)] were added to 200 μL of a stirred PBS solution at 37 °C, with continuous stirring for 3 h. The remaining of the starting derivatives were continuously assayed using HPLC analytical conditions as described above.

### Stability of 5′-O-trialkylsilylated decitabines in phosphate-buffered saline

Five microliters of DAC derivatives (1.0 mg/100 μL of DMSO) was added to 200 μL of a stirred PBS solution at 37 °C. The mixture was stirred continuously for 1 day. The remaining of the starting derivatives was assayed using the HPLC analytical conditions as described above.

### Cell lines and drug treatment

Human colon cancer cell lines (RKO, SW480, HT29, and HCT116), breast cancer cell lines (MDA-MB-231 and MCF7), sarcoma cell lines (MG-63, U-2 OS, Saos-2, HOS, and 143B), and a neuroblastoma cell (KELLY) were obtained from the American Type Culture Collection (ATCC; Manassas, VA). A gastric cancer cell line (TMK1), was kindly provided by Dr. W. Yasui at Hiroshima University. A neuroblastoma cell line (NB-1) was purchased from the Japanese Collection of Research Bioresources (Tokyo, Japan). A derivative cell line of HCT116, HML58-3, was established to detect DNA-demethylating agents [16]. Cells were checked for *Mycoplasma* infection using the MycoAlert mycoplasma detection kit (Lonza; Basel, Switzerland). The cells were harvested and kept frozen at − 80 °C until the extraction of genomic DNA and total RNA.

### Luminescence measurement of treated cells

Cells were seeded at a density of 5 × 10^3^ cells/well in a 96-well microplate on day 0 in triplicate, and 5 × 10^4^ cells/well in a six-well plate on day 0. On day 1, the medium was replaced with a medium containing a specific concentration of a drug, which was freshly dissolved in DMSO and filtered through a 0.2-μ filter. On day 5, the supernatant from each well of the 96-well microplate was collected to measure luminescence. Luminescence was measured using the Ready-To-Glow^TM^ Secreted Luciferase Reporter Assay (Clontech; Mountain View, CA) and a multimode plate reader ARVO^TM^MX (PerkinElmer Japan Co Ltd.; Kanagawa, Japan).

### Western blot analysis

Cells were seeded at a density of 3 × 10^5^ cells/10-cm dish and treated with appropriate drug(s) 24 h after the initial seeding. On day 5, the cells were lysed in RIPA buffer (20 mM Tris-HCl, pH 7.6; 150 mM NaCl; 1 mM EDTA; 0.1% SDS; and 1% NP-40) containing 3 mM dithiothreitol, proteinase inhibitor cocktail (Nacalai Tesque; Kyoto, Japan). Proteins were resolved by SDS-PAGE and transferred to an Immobilon-P nylon membrane (Merck Millipore; Billerica, MA). After blocking, each membrane was incubated with rabbit anti-DNMT1 antibody (Abcam, 1:1000) or mouse anti-PARP antibody (BD, 1:1000). Following three cycles of 10-min washes with PBS supplemented with 0.5% Tween 20 (PBS-T), blots were incubated with secondary antibodies (rabbit IgG, 1:1000; mouse IgG, 1:10000) and rewashed. Chemiluminescence was detected using an ECL kit (Biological Industries).

### Quantitative methylation-specific PCR

One microgram of *Eco*RV-digested genomic DNA was treated with sodium bisulfite as described previously [16]. Bisulfite-treated DNA was resuspended in 40 μL of TE-buffer (10 mM Tris, pH 8.0; 1 mM EDTA, pH 8.0), and 1 μL was used for quantitative methylation-specific PCR (qMSP) using primers specific to methylated and unmethylated target loci, including *MetLuc-UCHL1* promoter, *OSR2* promoter [18], and *UCHL1* promoter (Additional file 1: Table S1). As a fully unmethylated DNA control, genomic DNA from peripheral blood cells of a healthy male was amplified twice using a GenomiPhi DNA amplification kit (GE Healthcare Life Sciences; Little Chalfont, UK). As a fully methylated DNA control, the fully unmethylated DNA was methylated by *Sss*I methylase (New England Biolabs; Beverly, MA).

### Genome-wide DNA methylation analysis

Genome-wide DNA methylation analysis was performed using an Infinium Human MethylationEPIC BeadChip (Infinium EPIC; Illumina; Sam Diego, CA) as described previously [23, 24]. The BeadArray assessed the degree of methylation of 862,927 CpG probes as beta-values that ranged from 0 (unmethylated) to 1 (fully methylated). To reduce the data size and obtain data that were easy to handle, the CpG probes were grouped into 548,546 genomic blocks (GBs), which consisted of probes within 500 bp [23].

### Cell growth assay and viability assay

Cells were seeded at a density of 1 × 10^3^ to 5 × 10^3^ cells/well in a 96-well microplate or 1 × 10^4^ to 5 × 10^4^ cells/well in a six-well microplate on day 0 in triplicate, followed by treatment with drugs on day 1. The cell growth of HCT116 was estimated using IncuCyte (Essen BioScience, K.K.; Tokyo, Japan) for 94 h. The cell viability of SW480, HT29, and TMK1 was evaluated using a WST-8 [2-(2-methoxy-4-nitrophenyl)-3-(4-nitrophenyl)-5-(2, 4-disulfophenyl)-2H tetrazolium, monosodium salt] assay (Nacalai Tesque Inc., Kyoto, Japan). The numbers of cells of RKO, breast cancer cell lines, sarcoma cell lines, and neuroblastoma cell lines were counted using a microscope. The half-maximum inhibitory concentration (IC_50_) was obtained using the non-linear regression analysis of log (inhibitor) versus the normalized response with a variable slope using the GraphPad Prism software (GraphPad Software; La Jolla, CA).

### Quantitative reverse transcription PCR

Complementary DNA (cDNA) was synthesized from DNase-treated total RNA (1 μg) using oligo-(dT) 20 (Thermo Fisher Scientific; Waltham, MA) and SuperScript IV Reverse Transcriptase (Thermo Fisher Scientific). The number of cDNA molecules was quantified using qRT-PCR. The primer sequences and PCR conditions are shown in Additional file 1: Table S1. The copy number of each sample was calculated by comparing the amplification curve with those of standard DNA samples with known copy number. The number of target cDNA molecules was normalized to that of *GAPDH* cDNA molecules.

### Expression microarray experiments and data processing

Cy3-labeled cRNA was synthesized from 100 ng of total RNA using a Low Input Quick Amp Labeling Kit, One-Color (Agilent Technologies, Santa Clara, CA), and 600 ng of labeled cRNA was fragmented and hybridized to SurePrint G3 Human Gene Expression 8 x 60K v3 Microarray (Agilent Technologies). The hybridized microarray was scanned with an Agilent G2600D microarray scanner (Agilent Technologies). The scanned data were processed using Feature Extraction software (Agilent Technologies) and analyzed using GeneSpring software (Agilent Technologies). The 75th percentile of the signal intensity of all probes was normalized to be 0, and the genes with signal intensities of − 1 or more were regarded as showing positive expression.

### Ingenuity Pathways Analysis

Analysis of the biological functions of the microarray data was performed using Ingenuity Pathway Analysis (IPA) (Qiagen, Ingenuity H Systems, Redwood City, CA; https://www.qiagenbioinformatics.com/products/ingenuity-pathway-analysis/). The gene sets were prepared from the microarray data by selecting genes whose expression was induced (≥ twofold or more) by drug treatment and that had abundant expression (signal intensity > − 1 or more). By performing the core analysis, canonical pathways were algorithmically generated from the gene set.

### Pharmacokinetics in mice

Six-week-old female ICR mice were intraperitoneally injected with 0.20 mg/kg of DAC, 0.29 mg/kg of OR-2003, 0.32 mg/kg of OR-2009, 0.30 mg/kg of OR-2100, and 0.31 mg/kg of OR-2102. The doses were decided so that equivalent amounts of molar concentration would be administered. The blood plasma was collected before and at 5, 15, 30, 45, 60, 90, and 120 min after the treatment. All experimental procedures were approved by the Committee of National Cancer Center.

### Xenograft tumor formation assay in nude mice

HCT116 cells (5.0 × 10^6^ cells per mouse) were subcutaneously injected into the inguinal region of 6-week-old female nude mice (*n* = 81) (BALB/c-nu/nu; CLEA Japan, Tokyo, Japan). Once tumors reached an average volume of 80 mm^3^, mice bearing xenograft tumors were randomly divided into nine groups and treated intraperitoneally with vehicle (5% DMSO in 10% 2-hydroxypropyl-*β*-cyclodextrin solution), DAC, OR-2003, or OR-2100. The length and width of tumors were measured with standard calipers twice per week, and tumor volumes were calculated using the formula tumor volume = (length × width^2^) × 0.5. Body weight was measured twice per week. At 14 days after drug treatment, three mice, which showed the second, the fifth, and the eighth largest tumor volume, were selected as group 2, and at 18 days after the treatment, tumors were collected to analyze the DNA demethylation effect in vivo*.* The remaining mice were categorized as group 1 and continuously treated with drugs. At 29 days after the treatment, tumors, major organs, bone marrow, and blood serum were collected from six mice and blood count and biochemical tests were performed. The mice were maintained under standard conditions according to the institutional guidelines for animal care.

### Statistical analysis

Differences in the DNA demethylation level, gene expression level, cell viability, tumor volume, body weight, blood count, and the data of biochemical tests were analyzed using an unpaired Student’s *t* test. The results were considered significant when a *P* value < 0.05 was obtained by two-sided tests. All calculations were performed using the Microsoft Excel software (Microsoft Corp.; Seattle, WA).

## Additional file

Additional file 1: **Table S1.** Primers for qMSP and RT-qPCR. **Figure S1.** Screening of 35 synthesized compounds. (A) The DNA-demethylating activity of four 5′-O-trialkylsilylated AZAs and one 5′-O-trialkylsilylated DAC was screened under the drug treatment schedule on days 1 and 3. All 5′-O-trialkylsilylated AZAs exhibited a very low luciferase activity. (B) The DNA-demethylating activity of 11 5′-O-trialkylsilylated AZAs and 19 5′-O-trialkylsilylated DACs was screened under the drug treatment schedule on day 1. All 5′-O-trialkylsilylated AZAs displayed a considerably lower luciferase activity than that exhibited by DAC. Eleven 5′-O-trialkylsilylated DACs were selected for further study as their luminescence levels were at 1.0 × 10^6^ cps or more using 1.0 μM concentration. AZA, azacitidine; cps, counts/photons per second; DAC, decitabine. **Figure S2.** Primer positions for methylation analysis of the marker region. Primers were designed to analyze the level of DNA demethylation of the exogenous *UCHL1* promoter specifically. Since the reverse primer for the unmethylated DNA and the forward primer for the methylated DNA were located in the *MetLuc* sequence, we were able to distinguish between the exogenous and endogenous *UCHL1* promoters. **Figure S3.** Blood counts and chemistry. No differences in the number of red blood cells and in the level of platelets, hemoglobin, hematocrit, aspartate aminotransferase (AST), and blood urea nitrogen (BUN) were observed among the groups treated with DAC, OR-2003, and OR-2100. Results are shown as mean ± SD (*n* = 5 or 6/group). DAC, decitabine; SD, standard deviation. (DOCX 1253 kb)

## Data Availability

The data generated and/or analyzed during the current study are available in the GEO repository (GSE134293 for DNA methylation data and GSE134294 for expression array data).

## Abbreviations

AML: Acute myeloid leukemia
AUC: Area under the curve
AZA: Azacitidine
CDA: Cytidine deaminase
DAC: Decitabines
DMSO: Dimethyl sulfoxide
DNMT1: DNA methyltransferase 1
EGFP: Enhanced green fluorescent protein
MDS: Myelodysplastic syndrome
PBS: Phosphate-buffered saline
qMSP: Quantitative methylation-specific PCR
qRT-PCR: Quantitative reverse transcription PCR
SD: Standard deviation
WBC: White blood cell
