# Supplementary material for: Novel prodrugs of decitabine with greater metabolic stability and less toxicity
Source: Clin Epigenetics. 2019 Aug 1;11:111. doi: 10.1186/s13148-019-0709-y (PMC6670186; doi:10.1186/s13148-019-0709-y)
Supplement: Supplementary file 1 — Table S1. Primers for qMSP and RT-qPCR. Figure S1. Screening of 35 synthesized compounds. (A) The DNA-demethylating activity of four 5′-O-trialkylsilylated AZAs and one 5′-O-trialkylsilylated DAC was screened under the drug treatment schedule on days 1 and 3. All 5′-O-trialkylsilylated AZAs exhibited a very low luciferase activity. (B) The DNA-demethylating activity of 11 5′-O-trialkylsilylated AZAs and 19 5′-O-trialkylsilylated DACs was screened under the drug treatment schedule on day 1. All 5′-O-trialkylsilylated AZAs displayed a considerably lower luciferase activity than that exhibited by DAC. Eleven 5′-O-trialkylsilylated DACs were selected for further study as their luminescence levels were at 1.0 × 10^6^ cps or more using 1.0 μM concentration. AZA, azacitidine; cps, counts/photons per second; DAC, decitabine. Figure S2. Primer positions for methylation analysis of the marker region. Primers were designed to analyze the level of DNA demethylation of the exogenous UCHL1 promoter specifically. Since the reverse primer for the unmethylated DNA and the forward primer for the methylated DNA were located in the MetLuc sequence, we were able to distinguish between the exogenous and endogenous UCHL1 promoters. Figure S3. Blood counts and chemistry. No differences in the number of red blood cells and in the level of platelets, hemoglobin, hematocrit, aspartate aminotransferase (AST), and blood urea nitrogen (BUN) were observed among the groups treated with DAC, OR-2003, and OR-2100. Results are shown as mean ± SD (n = 5 or 6/group). DAC, decitabine; SD, standard deviation. (DOCX 1253 kb) [file 13148_2019_709_MOESM1_ESM.docx]

# Supplementary Materials

**Novel prodrugs of decitabine with greater metabolic stability and less toxicity**

Naoko Hattori, Magoichi Sako, Kana Kimura, Naoko Iida, Hideyuki Takeshima, Yoshitaka Nakata, Yutaka Kono, Toshikazu Ushijima

## Supplementary Information

Cytidine deaminase (CDA) catalyzes the hydrolytic deamination at the C4-position of cytidine and its nucleoside analogs include cytosine arabinoside (cytarabine or ara-C) and 5-azacytidine (AZA). Their Michaelis constants are 11 µM for cytidine, 26 µM for 2'-deoxycytidine, 30 µM for 5-fluorocytidine, 88 µM for ara-C, and 430 µM for AZA. This enzyme has no activity towards the hydrolytic deamination of cytidine nucleotides and its 2'-deoxynucleotides, which are 5'-O-phosphate analogs of cytidine nucleosides and their 2'-deoxynucleosides [J. Clinical Investigation, 1974, 53(3), 922-931].

2'-Deoxy-5-azacytidine (decitabine; DAC) is sensitive to CDA, though the structures of products in this reaction are not clear yet. We have previously found that 5'-O-triethylsilylated derivatives of AZA and DAC were stable (most unchanged derivatives are retained even after 0.5 hr) in the presence of CDA under conditions where the unmodified DAC was completely degraded within 0.5 hr (Table 1 in the main text). Therefore, we have investigated the possibility of 5'-O-trialkylsilylated derivatives as prodrugs for AZA and DAC.

The trialkylsilyl functional groups are very convenient and useful for the protection of hydroxyl groups in the chemical synthesis of biologically active compounds. Their chemical stability depends on the bulkiness of alkyl groups on the silicon atom. In general, their stability towards acidic media increases as indicated: Me_3_Si-OR (1) < Et_3_Si-OR (64) <<< ^tert^BuMe_2_Si-OR (20,000) << (^iso^Pr)_3_Si-OR (700,000) << ^tert^BuPh_2_Si-OR (5,000,000), and their stability towards basic media increases in the following order: Me_3_Si-OR (1) < Et_3_Si-OR (10 - 100) <<< ^tert^BuMe_2_Si-OR - ^tert^BuPh_2_Si-OR (20,000) << (^iso^Pr)_3_Si-OR (100,000) ["Greene's Protective Groups in Organic Synthesis", 5th Ed.; Wuts, P.G.M., 2014, pp.201-270]. In fact, the 5'-O-trimethylsilyl derivative of DAC is unstable in a protic solvent and cannot be isolated in a pure state. On the other hand, its 5'-O-^tert^BuMe_2_silyl derivative is stable, even in a protic solvent, and can be easily prepared.

In view of these observations, we have synthesized a variety of 5'-O-trialkylsilyl derivatives of AZA and DAC. Among these derivatives, the following Table summarizes the partition coefficient (log *P*_n-Octanol/PBS_) of the 5'-O-trialkylsilylated DAC and their stability in phosphate-buffered saline (PBS) solution at 37°C.


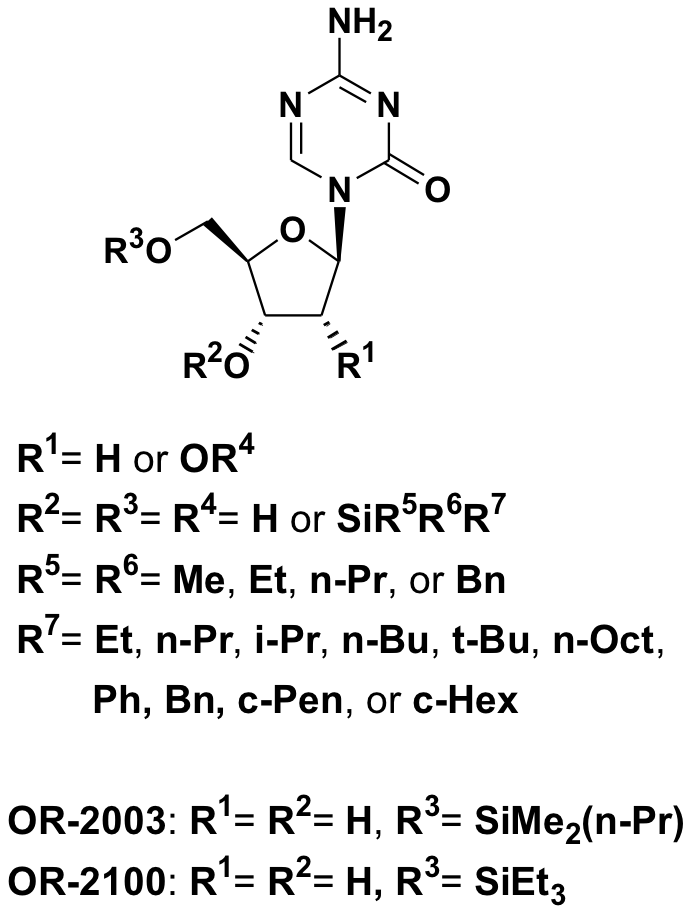


R^1^ = R^2^= H

R^3^ = SiR^5^R^6^R^7^

R^5^ = R^6^ = Methyl, Ethyl, n-Propyl, or Benzyl

R^7^ = Methyl, Ethyl, n-Propyl, i-Propyl, n-Butyl, t-Butyl, n-Octyl, Benzyl, c-Pentyl, or c-Hexyl

Table. Structure, stability, and Log *P* value of 5'-O-trialkylsilylated DACs

| R^5^ | R^6^ | R^7^ | t_1/2_ in PBS | Log *P*_O/PBS_ | Compound |
| --- | --- | --- | --- | --- | --- |
| Methyl | Methyl | Methyl | Unstable | - |  |
| Methyl | Methyl | Ethyl | 0.9 hr | - |  |
| Methyl | Methyl | n-Propyl | 1.0 hr | 1.64 | OR-2003 |
| Methyl | Methyl | n-Butyl | 1.3 hr | 4.56 |  |
| Methyl | Methyl | n-Octyl | 1.5 hr | - |  |
| Methyl | Methyl | Benzyl | 2.5 hr | 1.73 |  |
| Methyl | Methyl | i-Propyl | 12 hr | 1.48 |  |
| Methyl | Methyl | c-Pentyl | 15 hr | 2.16 |  |
| Methyl | Methyl | c-Hexyl | 14 hr | 2.52 | OR-2009 |
| Methyl | Methyl | t-Butyl | > 24 hr | 1.91 |  |
| Ethyl | Ethyl | Methyl | 4 hr | 1.49 |  |
| Ethyl | Ethyl | Ethyl | 16 hr | 2.14 | OR-2100 |
| Ethyl | Ethyl | n-propyl | 19 hr | 2.54 | OR-2102 |
| Ethyl | Ethyl | i-Propyl | > 24 hr | 2.56 |  |
| Ethyl | Ethyl | c-Pentyl | > 24 hr | 3.10 |  |
| n-Propyl | n-Propyl | n-Propyl | 22 hr | 5.20 |  |
| Benzyl | Benzyl | Benzyl | > 24 hr | 4.69 |  |

The Table indicates that the 5'-O-silyl derivatives not having a bulky alkyl group on the silicon atom, such as EtMe_2_Si-, n-PrMe_2_Si-, and n-BuMe_2_Si-, are smoothly hydrolyzed (t_1/2_ = ca. 1 hr - 2 hr), even under mild conditions, to give DAC almost quantitatively, and that the 5'-O-silyl derivatives having a bulky alkyl group on the silicon atom, such as i-PrMe_2_Si-, c-PenMe_2_Si-, and Bn_3_Si-, are gradually hydrolyzed (t_1/2_ = ca. 10 hr - > 24 hr) under analogous conditions to give DAC. On the enzymatic stability of the 5'-O-silylated DAC, the following results were obtained: the 5'-O-silyl derivatives having a small and not so bulky alkyl group on the silicon atom, such as n-PrMe_2_Si-, i-PrMe_2_Si-, and MeEt_2_Si-, are stable in the presence of CDA but the 5'-O-silyl derivatives having bulky alkyl group on the silicon atom, such as BnMe_2_Si- and n-PrEt_2_Si-, are gradually degraded by CDA.

On the basis of the above facts, we have selected a 5'-O-(n-PrMe_2_)silyl derivative (OR-2003), a 5'-O-(c-HexMe_2_)silyl derivative (OR-2009), a 5'-O-(Et_3_)silyl derivative (OR-2100), and a 5'-O-(n-PrEt_2_)silyl derivative (OR-2102) as having an orally druggable lipophilicity (log *P* = +1.0 - +3.0) and proper stability in the presence or absence of CDA to estimate their abilities as a prodrug for DAC, in this paper.

## Table S1. Primers for qMSP and RT-qPCR

* The primers were designed on the bottom strand DNA after bisulfite modification.

| **Experiment** | **Name** | **Primer sequence** | | **Anealing**  **temp. (˚C)** |
| --- | --- | --- | --- | --- |
|  |  | **Forward** | **Reverse** |  |
| **qMSP** | *UCHL1_MetLuc*_M*** | CGGGTTCGCGGTATCGTC | TACCCATCTAACCGCGACCG | 57 |
|  | *UCHL1_MetLuc*_U | GGTTTGTATTTATTTGGTTGTGATT | CCTTCCAAACCCACAATATCA | 52 |
|  | *OSR2*_M | CGTAGCGCGTGGGATTTTAC | CCAATACTAACAAACCGAAACG | 57 |
|  | *OSR2*_U | GGTTTAGGAGGATGAAGTGT | CACCCTATAACCACCTTTCCCACA | 57 |
|  | *UCHL1*_M | TCGTATTTATTTGGTCGCGATC | CTATAAAACGCCGACCAAACG | 64 |
|  | *UCHL1*_U | GGTTTGTATTTATTTGGTTGTGATT | CAACTATAAAACACCAACCAAACA | 61 |
| **RT-qPCR** | *GAPDH* | AGGTGAAGGTCGGAGTCAACG | AGGGGTCATTGATGGCAACA | 58 |
|  | *UCHL1* | TCCGCTAGCTGTTTTTCGTC | GGTGAACACCACCTTGATGTC | 58 |
|  | *SFRP1* | TGAAATCTGAGGCCATCATT | TTCAGGGGCTTCTTCTTCTT | 58 |


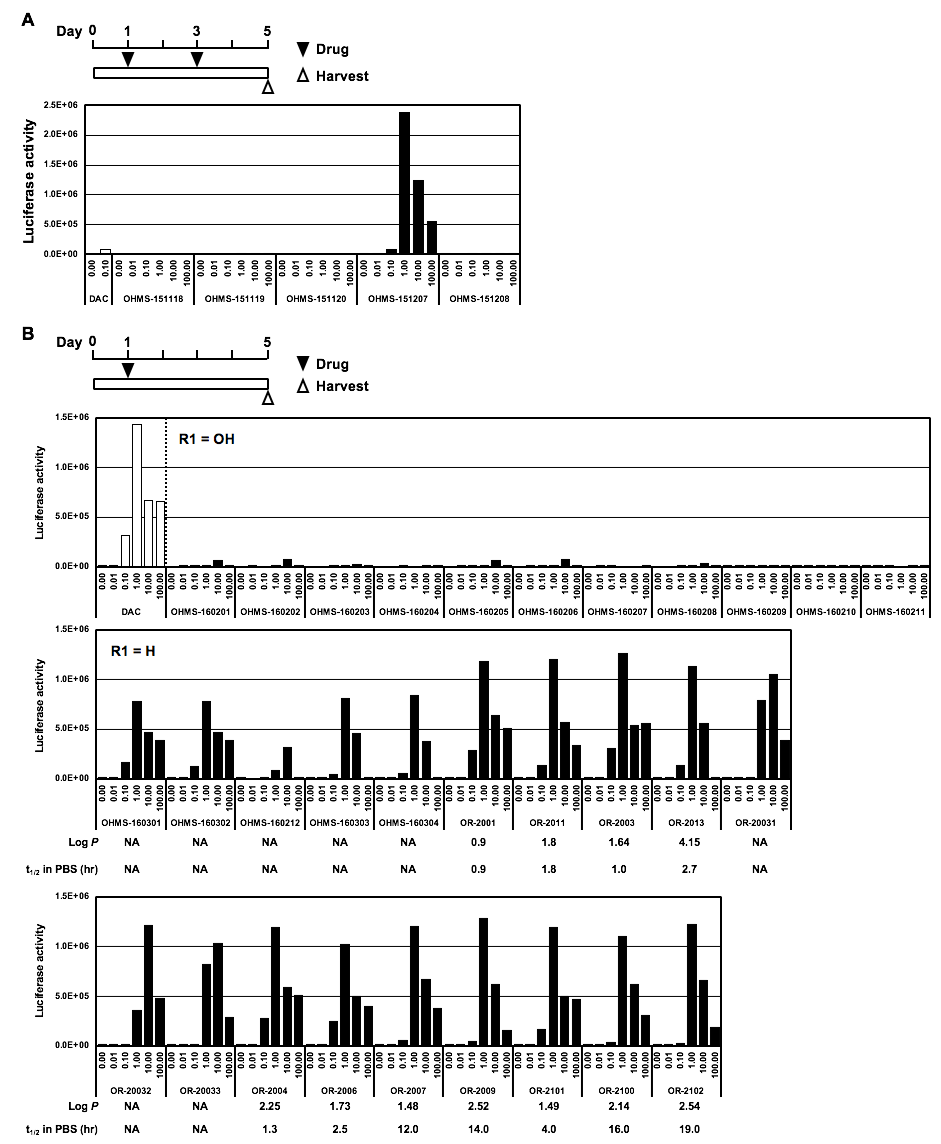


## Figure S1

Screening of 35 synthesized compounds. (A) The DNA demethylating activity of four 5'-O-trialkylsilylated AZAs and one 5'-O-trialkylsilylated DAC was screened under the drug treatment schedule on Days 1 and 3. All 5'-O-trialkylsilylated AZAs exhibited a very low luciferase activity. (B) The DNA demethylating activity of 11 5'-O-trialkylsilylated AZAs and 19 5'-O-trialkylsilylated DACs was screened under the drug treatment schedule on Day 1. All 5'-O-trialkylsilylated AZAs displayed a considerably lower luciferase activity than that exhibited by DAC. Eleven 5'-O-trialkylsilylated DACs were selected for further study as their luminescence levels were at 1.0 × 10^6^ cps or more using 1.0 µM concentration. AZA, azacitidine; cps, counts/photons per second; DAC, decitabine.


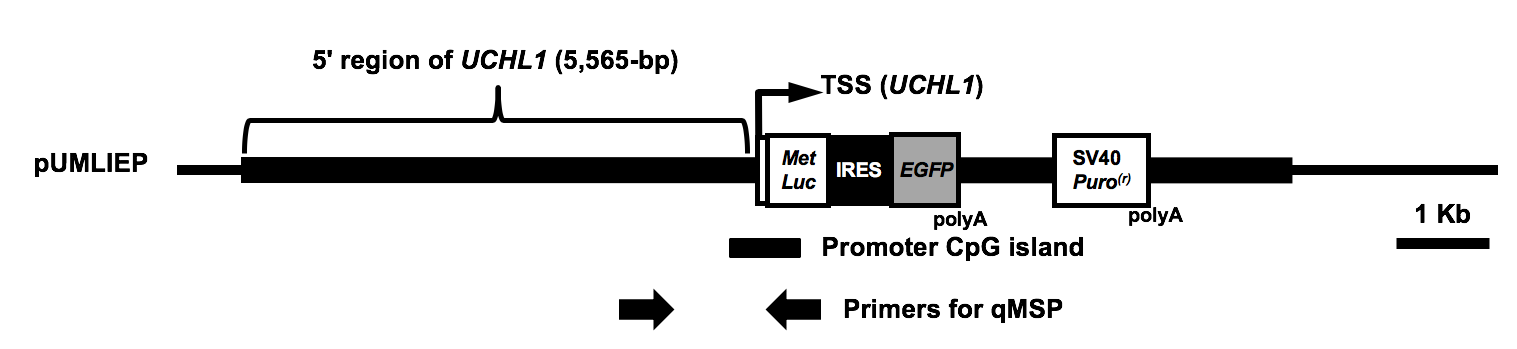


## Figure S2

Primer positions for methylation analysis of the marker region. Primers were designed to analyze the level of DNA demethylation of the exogenous *UCHL1* promoter specifically. Since the reverse primer for the unmethylated DNA and the forward primer for the methylated DNA were located in the *MetLuc* sequence, we were able to distinguish between the exogenous and endogenous *UCHL1* promoters.


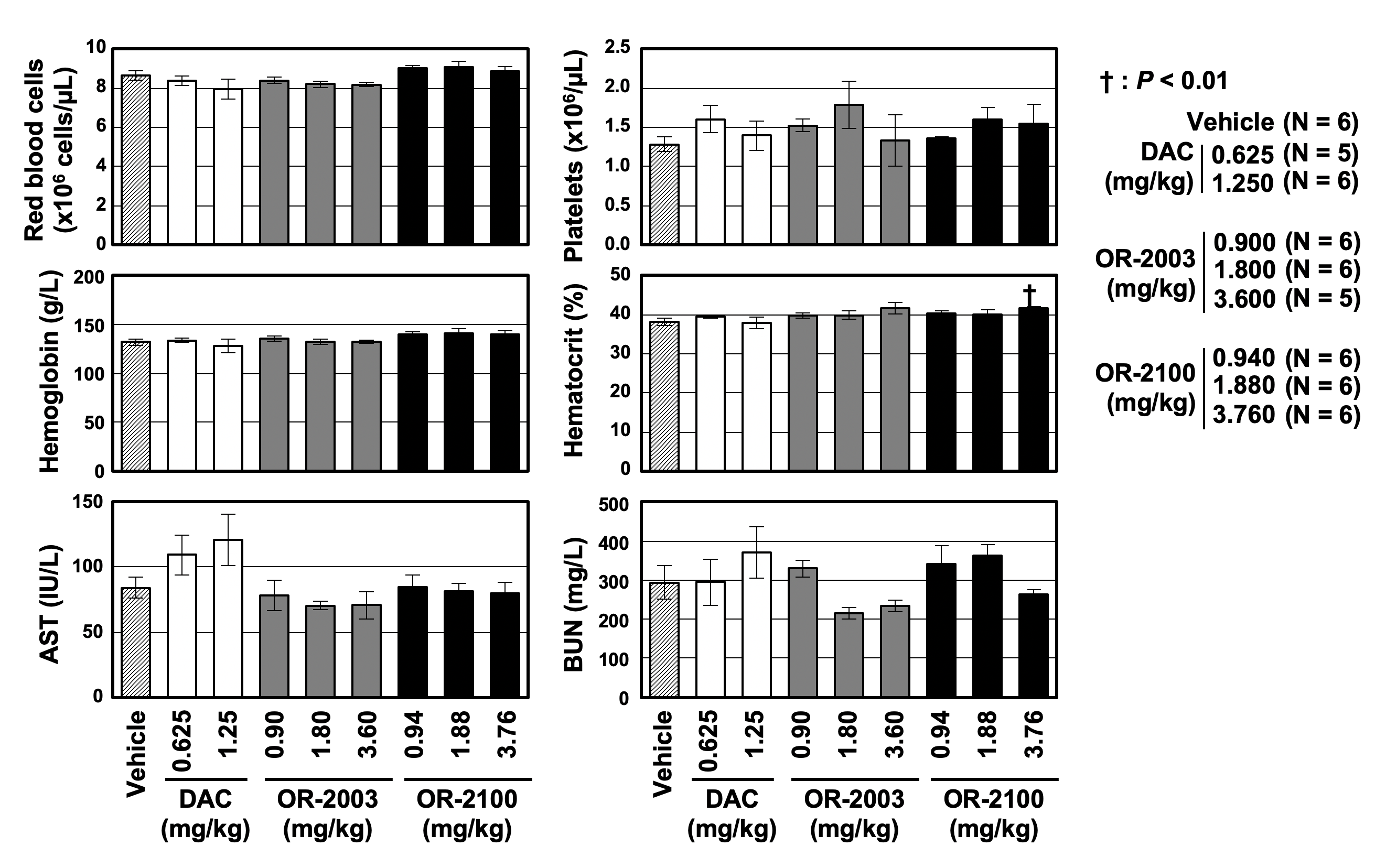


## Figure S3

Blood counts and chemistry. No differences in the number of red blood cells and in the level of platelets, hemoglobin, hematocrit, aspartate aminotransferase (AST), and blood urea nitrogen (BUN) were observed among the groups treated with DAC, OR-2003, and OR-2100. Results are shown as mean ± SD (*n* = 5 or 6/group). DAC, decitabine; SD, standard deviation.
